# Harnessing Machine Learning to Enhance Transition State Search with Interatomic Potentials and Generative Models

**DOI:** 10.1002/advs.202506240

**Published:** 2025-07-13

**Authors:** Qiyuan Zhao, Yunhong Han, Duo Zhang, Jiaxu Wang, Peichen Zhong, Taoyong Cui, Bangchen Yin, Yirui Cao, Haojun Jia, Chenru Duan

**Affiliations:** ^1^ Deep Principle Inc. Cambridge MA 02139 USA; ^2^ AI for Science Institute Beijing 100080 P. R. China; ^3^ DP Technology Beijing 100080 P. R. China; ^4^ Academy for Advanced Interdisciplinary Studies Peking University Beijing 100871 P. R. China; ^5^ Bakar Institute of Digital Materials for the Planet UC Berkeley California 94720 United States; ^6^ Department of Chemistry Tsinghua University Beijing 100084 China; ^7^ Frontiers Science Center for Transformative Molecules School of Artificial Intelligence Shanghai Jiao Tong University Shanghai 200240 China

**Keywords:** automated reaction prediction, diffusion model, machine learning interatomic potential, reaction networks, transition states

## Abstract

Transition state (TS) search is crucial for illuminating chemical reaction mechanisms but remains the major bottleneck in automated discovery because of the high computational cost. Recently, machine learning interatomic potentials (MLIPs) and generative models have shown promise in accelerating TS search, but their comparative strengths and limitations remain unclear. In this study, the first systematic and rigorous benchmarking framework is established to evaluate the effectiveness of ML methods in TS search, enabling a standardized and application‐relevant assessment of their performance. Using an end‐to‐end TS search workflow, seven representative MLIPs are benchmarked alongside React‐OT, a state‐of‐the‐art generative model. These results demonstrate that pre‐trained foundation MLIPs frequently fall short in reliably localizing TSs without task‐specific fine‐tuning. Furthermore, traditional energy and force metrics alone do not reliably predict TS search success, underscoring the need for more tailored evaluation criteria. Notably, with the same graph neural network architecture, React‐OT frequently outperforms its MLIP counterpart, highlighting the potential of generative approaches for TS discovery. This benchmark serves as a critical foundation for the development and evaluation of future ML methods in chemical reactions, offering guidance for improving their generalizability and reliability in reactive chemistry.

## Graphical abstract

Comprehensive evaluation of MLIPs and generative models for TS search using an end‐to‐end benchmark platform. Success rates, TS quality, and barrier prediction errors are systematically compared across models. The results reveal key performance trade‐offs and provide actionable insights for improving ML‐based transition state search and reaction exploration

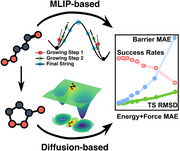

## 1 Introduction

Computational transition state (TS) characterization plays a critical role in distinguishing between competing reaction pathways, elucidating reaction mechanisms, and predicting selectivity, making it an essential tool in computational chemistry across a wide range of applications.^[^ ^1^, ^2^, ^3^, ^4^, ^5^, ^6^, ^7^, ^8^, ^9^, ^10^ ^]^ Recently, the growing demand for novel drugs and materials has surged the need for fast and accurate TS searches,^[^ ^11^ ^]^ as they are crucial for efficiently discovering new reactions, exploring reaction networks, and identifying promising pathways for further development.^[^ ^12^, ^13^, ^14^ ^]^ Very few characterization techniques, however, can unravel elusive TS properties or their structures in experiments due to the transient nature of TS.^[^ ^15^, ^16^ ^]^ Moreover, locating TS is up to two orders of magnitude more computationally expensive than searching for the minimum energy geometries of individual molecules, as saddle point optimization is more complex and requires not only energy and force but also Hessian evaluations.^[^ ^17^, ^18^ ^]^ This makes TS search the bottleneck in large‐scale automated reaction prediction. To overcome these high computational requirements, machine learning (ML) approaches have been increasingly adopted in recent years to accelerate TS search by reducing reliance on DFT calculations.^[^ ^13^, ^19^, ^20^, ^21^, ^22^, ^23^, ^24^ ^]^ These approaches can be broadly categorized into two main strategies. The first involves developing machine learning interatomic potentials (MLIPs) that accurately describe the potential energy surface (PES), particularly the region near the minimum energy path (MEP).^[^ ^25^, ^26^ ^]^ By enabling the efficient calculation of energy, force, and Hessian, MLIPs integrate seamlessly with physics‐based transition state search algorithms, significantly speeding up the process while maintaining high accuracy.^[^ ^27^ ^]^ The second strategy focuses on ML‐based transition state generation methods, which aim to directly predict transition states without relying on exhaustive PES exploration methods.^[^ ^28^, ^29^, ^30^, ^31^, ^32^ ^]^

MLIPs have emerged as promising tools for modeling materials and molecules.^[^ ^26^, ^33^, ^34^, ^35^, ^36^, ^37^, ^38^ ^]^ Classic MLIPs established foundational frameworks using geometric descriptors embedded in fully connected neural networks, such as ANI,^[^ ^39^, ^40^, ^41^ ^]^ Deep Potential,^[^ ^42^ ^]^ and the DPA‐2 model.^[^ ^43^ ^]^ More recently, a wide range of equivariant graph neural networks (EGNNs) have been proposed, demonstrating state‐of‐the‐art performance in energy and force predictions, including models such as PaiNN,^[^ ^44^ ^]^ MACE,^[^ ^45^ ^]^ Equiformer,^[^ ^46^, ^47^ ^]^ and LEFTNet.^[^ ^48^ ^]^ While these models have demonstrated success in modeling equilibrium systems, their application to reactive systems – characterized by bond breaking and forming – remains under‐explored yet essential. Although earlier reactive MLIPs were largely restricted to specific reactions, such as S_*N*_2 and pericyclic reactions,^[^ ^49^, ^50^, ^51^, ^52^, ^53^ ^]^ recent progress has shown their potential in broader reactive chemistry.^[^ ^43^, ^54^, ^55^, ^56^, ^57^ ^]^ For example, Zhang et al.^[^ ^56^ ^]^ develop a general reactive MLIP (ANI‐1xnr) through automated sampling of condensed‐phase reactions. Schreiner et al. applied the PaiNN to the Transition1x dataset, which includes approximately 10 000 reactions and 10 million data points, demonstrating its capability in identifying transition states across a wide range of reactions.^[^ ^57^ ^]^ Furthermore, the Transition1x dataset is also utilized as one of several pre‐training datasets within the DPA‐2 framework, where diverse multidisciplinary datasets are collectively trained to create a unified descriptor. This approach helps improve the model's transferability and performance in reactive systems.^[^ ^43^ ^]^ Building on these advancements, reactive MLIPs are well‐positioned to expedite TS searches across a broad spectrum of reaction spaces, thereby enabling promising applications in the exploration of complex reaction networks and the identification of novel pathways.^[^ ^27^, ^58^, ^59^, ^60^, ^61^, ^62^, ^63^ ^]^

Methods that directly predict TS structures from reactant and product geometries have attracted growing interest as an alternative since they bypass the need for minimal energy pathway construction and optimization. These models have evolved from simply predicting geometric features of TS structures (e.g., interatomic distances) by using graph neural networks (GNNs)^[^ ^28^ ^]^ or transformers,^[^ ^32^ ^]^ to generative models that directly predict the TS structures.^[^ ^29^, ^30^, ^31^ ^]^ Under the framework of diffusion models, TSDiff generates the 3D geometry of TS directly from the 2D graphs or SMILES strings of reactants and products, reducing the cost of obtaining any 3D structures in a reaction a priori.^[^ ^30^ ^]^ OA‐ReactDiff^[^ ^29^ ^]^ learns the joint distribution of three distinct structures in an elementary reaction: reactants, TS, and products. Equipped with an object‐aware SE(3) GNN that preserves all required symmetries in chemical reactions, OA‐ReactDiff achieves state‐of‐the‐art performance on generated structure similarity. By further encoding a more chemically intuitive TS guess and adopting the optimal transport objective, React‐OT, which is tailored for double‐ended TS search, makes generative modeling deterministic and achieves more accurate TS generation with much lower (<10%) inference cost.^[^ ^31^ ^]^ Despite significant progress in applying machine learning to transition state (TS) search, quantifying the capability and accuracy of the two distinct approaches, MLIPs and generative models, remains challenging due to their inherent differences in design and application. Although both have shown promise in advancing TS searches, their performance across diverse reaction spaces necessitates a systematic evaluation. The emergence of powerful TS search tools, such as Yet Another Reaction Program (YARP)^[^ ^64^, ^65^ ^]^ and the autodE package,^[^ ^66^ ^]^ has led to breakthrough performance across a wide range of chemical systems, opening new opportunities for standardized and rigorous comparisons.

In this work, we provide a comprehensive evaluation of MLIPs and generative models for TS search in an end‐to‐end fashion, leveraging the YARP framework, to fill this gap. Seven MLIPs, spanning a wide range of architectures, were benchmarked: from early transferable models based on direct 3D geometry embedding (ANI‐1x),^[^ ^39^ ^]^ to those using more modern graph convolutional approaches (CHGNet);^[^ ^67^ ^]^ models built on EGNNs (LEFTNet,^[^ ^48^ ^]^ MatterSim^[^ ^68^ ^]^); foundation models incorporating pre‐training strategies for enhanced transferability (MACE‐OFF23);^[^ ^45^, ^69^, ^70^ ^]^ multi‐task pretraining approaches (DPA‐2),^[^ ^43^ ^]^ and the latest force‐predicting models with complex architectures (Orb).^[^ ^71^ ^]^ These are compared against React‐OT, a state‐of‐the‐art diffusion‐based generative model for TS generation. Through this comparison, we highlight the differing and often complementary strengths of these two model classes in reactive systems, and suggest promising directions for future model development, including hybrid strategies and task‐specific enhancements.

## 2 Results and Discussion

### 2.1 Transition State Search Workflow

**Figure 1:**
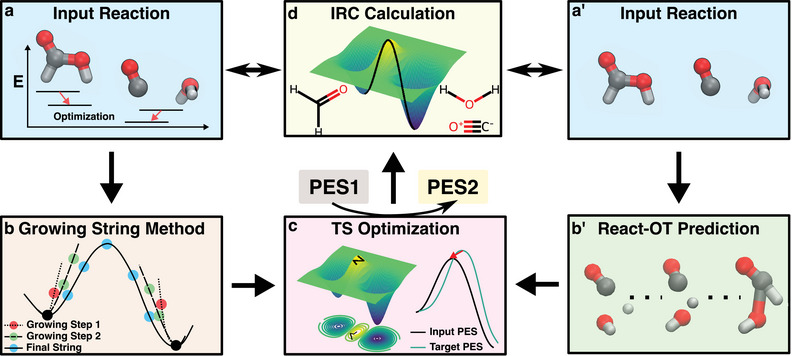
Overview of two approaches for transition state (TS) search: Machine‐Learning Interatomic Potentials (MLIPs) and React‐OT. a) Reactant and product geometries are optimized using MLIPs. b) The Growing String Method (GSM) constructs a minimal energy pathway at the MLIP potential energy surface (PES), providing an initial TS guess. c) Hessian‐based TS optimization refines the TS initial guess, guiding it to a saddle point (true TS) on the target PES. d) An intrinsic reaction coordinate (IRC) calculation validates the optimized TS by comparing the end points of the resulting trajectory with the initial reactant and product geometries (a and a'). a'‐b') In the React‐OT approach, a linear interpolation between reactant and product geometries is transformed into a TS structure, which is then optimized on the target PES (e.g., DFT).

The MLIP‐based TS search workflow consists of four key steps. First, MLIP‐level geometry optimization is performed on the input reactant and product (**Figure** **1**a). Next, a growing string method (GSM)^[^ ^72^ ^]^ calculation is initiated using the optimized geometries to construct a MEP, with energies and gradients evaluated by the corresponding MLIP (Figure 1b). The highest‐energy node, ideally near the saddle point on the potential energy surface (PES), serves as the TS initial guess and is refined using Hessian‐based restricted‐step rational‐function‐optimization (RS‐I‐RFO) optimization (TS optimization, Figure 1c).^[^ ^73^ ^]^ During each optimization step, the Hessians, computed from the derivative of MLIP forces, guide the TS search. Finally, an intrinsic reaction coordinate (IRC) calculation confirms that the identified TS corresponds to the expected reaction pathway by comparing the IRC endpoints with the input reactant and product (Figure 1d). If they match on both sides, the TS is called “intended”, indicating that the TS search has successfully located a TS along the expected reaction pathway. Otherwise, it is termed “unintended”, suggesting that the identified TS does not correspond to the anticipated reaction mechanism. Additionally, the TS optimization (Figure 1c) can transfer the TS structure between different levels of theory (e.g., from MLIP to DFT), under the same hypothesis that these two TS structures are close to each other.

In the generative AI‐based approach, the predicted TS structures are treated as TS initial guesses, equivalent to those obtained from converged GSM calculations. These structures are optimized using either DFT (ωB97X/6‐31G*, the level React‐OT and MLIPs are trained on) or a MLIP, converging to saddle points on the corresponding PES (Figure 1c). Finally, IRC calculations are performed to validate the obtained TSs (Figure 1d). Detailed descriptions of each component are provided in the Experimental Section.

### 2.2 Benchmarking Various MLIPs on a Reactive Dataset

The Transition1x dataset,^[^ ^74^ ^]^ which contains geometries sampled during nudged‐elastic band (NEB) calculations on 10 073 graphically‐enumerated reactions,^[^ ^75^ ^]^ was used to train and test MLIPs due to its diverse reaction space. The dataset was split following the same procedure as in the previous React‐OT study, with 9000 reactions allocated to the training set and 1073 reactions to the validation set. This consistent split enables further direct comparison between React‐OT and MLIP approaches. All geometries from the 9000 training reactions were assigned to the training set, and those from the 1073 validation reactions to the validation set, minimizing potential data leakage. Additionally, since the TSs in the Transition1x dataset are the highest‐energy nodes in the climbing image nudged elastic band method (CI‐NEB) string and have not been further optimized and verified, we performed TS optimization and IRC calculations (Figure 1c,d) at the same level of theory (ωB97X/6‐31G*). This process identified only 960 reactions with intended TSs. Consequently, all geometries obtained from 1073 validation reactions were used for testing energy and force predictions, while the 960 reactions with intended TSs were used for testing the TS search.

**Figure 2:**
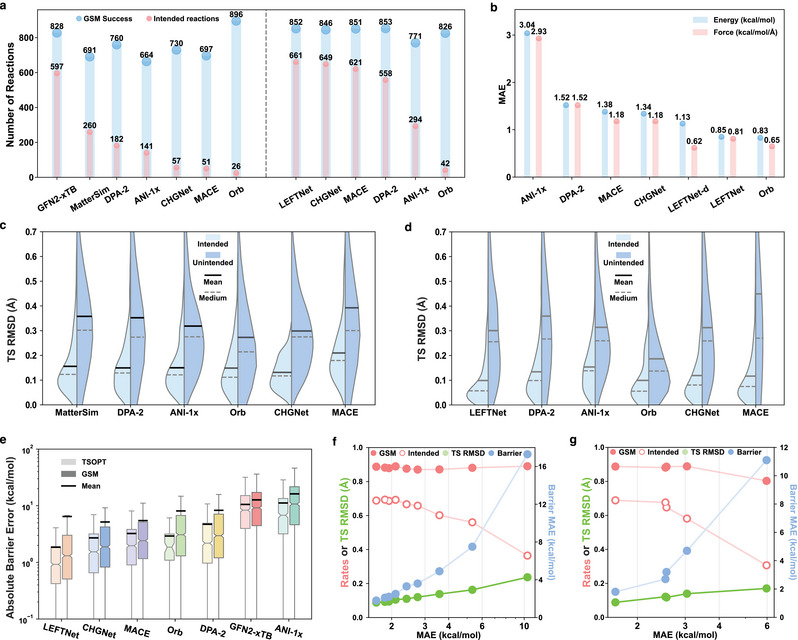
Comparison of seven different MLIPs and GFN2‐xTB on the Transition1x dataset. a) Number of reactions with a converged GSM pathway (blue) and intended TS (red) when testing the TS search workflow (Figure 1a–d) on the Transition1x withheld validation set with different MLIPs and GFN2‐xTB as the computational engines. Left and right plots show results before and after training on the Transition1x, respectively. b) Mean absolute error (MAE) of energies and forces on the Transition1x validation set after training. c,d) Violin plots showing the distribution of root‐mean‐square deviations (RMSD) between GSM‐obtained TSs and DFT‐optimized TSs. The left (lighter colors) and right (darker colors) parts of each violin represent intended and unintended TSs, respectively, after optimization and validation (Figure 1c,d) on the same PES. Panels c and d illustrate the performance before and after training on the Transition1x, respectively. e) Box plots showing the distribution of absolute errors for reaction barriers. Lighter colors (left) represent barriers computed by optimizing both reactants and TSs using the corresponding MLIPs, while darker colors (right) represent barriers directly obtained from GSM calculations. DFT‐level barriers serve as the reference. f,g) Correlation between explicit and implicit metrics. The explicit metric on the x‐axis is the direct summation of energy and force MAE values, with both having the same unit as panel (b). Implicit metrics on the y‐axis include GSM success rate (red solid circles), intended rate (red hollow circles), TS RMSD (green circles), and absolute barrier error (blue circles, using right y‐axis). Metrics are calculated from f) nine checkpoint files during LEFTNet training and g) five MLIPs after complete training on the Transition1x dataset (ordered left to right: LEFTNet, CHGNet, MACE, DPA‐2, ANI‐1x).

Six MLIPs (excluding LEFTNet) which have been extensively pre‐trained on non‐reactive datasets (i.e., without bond‐breaking or forming events) were first tested for TS search on the 960 validation reactions, along with the widely used semi‐empirical Geometry, Frequency, Noncovalent, eXtended Tight Binding (GFN2‐xTB) model. Two statistics were used to evaluate the accuracy of describing the PES around the minimal energy pathways (**Figure** **2**a, left): the number of reactions with a converged GSM pathway (denoted as GSM Success), and the number of reactions with the intended transition state (denoted as intended reactions). A lower GSM success rate indicates that the MLIP fails to provide reliable predictions at certain points on the PES. Besides, a higher intended rate reflects the MLIP's ability to accurately reproduce the DFT PES, including capturing the PES curvatures such that the TS could be accurately predicted. It is worth noting that while the Orb‐v2 model constructs the highest number of GSM pathways, the intended reaction rate is extremely low (∼2%). This discrepancy arises because the Orb model predicts forces directly, rather than derives them from energy gradients, which causes the loss of the energy conservation law,^[^ ^76^, ^77^ ^]^ and results in poor Hessians that are vital in locating TS structures. While MatterSim identifies the highest number of intended TSs across all MLIPs, its relatively low GSM success rate suggests that the pathway construction may be less reliable, with the model struggling to accurately capture the full MEP region. The DPA‐2 model, whose original version (2.3.1‐v3.0.0rc0) includes Transition1x data in the pre‐training step, was re‐trained with the Transition1x head removed for a fair comparison. The resulting “26‐head” DPA‐2 model achieves the second‐highest GSM success and intended rates, indicating strong transferability from non‐reactive to reactive regimes. The ANI‐1x and MACE‐OFF23 models, trained specifically on organic molecules, construct 664 and 697 GSM pathways and identify 141 and 51 intended TSs, respectively. Surprisingly, CHGNet, which is designed for and trained on inorganic bulk materials, outperforms MACE‐OFF23 in both GSM success and intended rates. Notably, none of the MLIPs outperform GFN2‐xTB. The GSM‐obtained TSs (i.e., the highest energy nodes) are classified as “intended” or “unintended” based on their outcome after TS optimization (Figure 1c) and intrinsic reaction coordinate (IRC) calculation (Figure 1d). If the TS successfully converges to an intended TS after both steps, it is labeled as “intended.” If it fails at either step or does not lead to the intended TS, it is labeled as “unintended.” RMSD between the GSM‐obtained and DFT‐optimized TSs exhibit distinct distributions for the “intended” and “unintended” categories, emphasizing the importance of providing an accurate initial TS guess in the TS search workflow. Interestingly, different MLIPs show relatively similar performance (mean and median RMSD) for “intended” TSs, with the exception of MACE‐OFF23, which shows slightly higher RMSDs. Overall, all pretrained universal MLIPs give an unsatisfactory performance on searching TS, especially when locating intended TS structures.

After training six MLIPs on the Transition1x data (excluding MatterSim, which does not support fine‐tuning), the MAEs of energies and forces on the validation set are shown in Figure 2b. The training curves, performance of atomic energy and force predictions of different MLIPs, are shown in Figures S2–S5 (Supporting Information). Details on the model architectures and training details can be found in the Experimental Section and Section S2 (Supporting Information), respectively. ANI‐1x exhibits the highest errors in both energy and force prediction (∼3 kcal/mol), likely due to its simpler architecture. Although DPA‐2 shows improved performance after exposure to reactive data, its validation MAEs are the second highest among all MLIPs. The CHGNet and MACE‐OFF23 models show similar performance, despite their distinct architectures. LEFTNet, though trained from scratch, achieves the lowest energy and force MAEs among the MLIPs that derive forces from energy gradients. In contrast, models that directly predict forces (LEFTNet‐d and Orb‐v2) achieve even lower force MAEs (0.62 and 0.65 kcal/mol/Å, respectively), with energy MAEs of 1.13 and 0.83 kcal/mol, respectively, demonstrating the advantages of the direct force prediction scheme in getting a lower MAE metric. These MLIPs were re‐evaluated on the 960 validation reactions (Figure 2a, right). LEFTNet, CHGNet, and MACE rank as the top‐3 MLIPs in identifying intended TSs, locating 661, 649, and 621 intended TSs, respectively, outperforming GFN2‐xTB (549 intended TSs) on this set of reactions. DPA‐2 and ANI‐1x perform worse, consistent with their higher MAE results. The Orb‐v2 model, while slightly improved, still yields a much lower intended rate (∼5%) due to the asymmetric Hessians used in the TS optimization step. The TS RMSDs show a similar trend as the intended rates, with LEFTNet having the lowest mean RMSD (0.10Å) and ANI‐1x having the highest (0.15Å).

All MLIPs were trained using the same level of theory (ωB97X/6‐31G*), allowing the barriers to be benchmarked against DFT‐calculated values (Figure 2e). In general, the absolute barrier errors for optimized TSs are much lower than those for GSM‐derived barriers. The improvement can be rationalized from (1) the enhanced quality of the optimized TSs, and (2) the TS optimization helps exclude unintended TSs. Such improvement emphasizes the importance of TS optimization and verification after GSM calculations. The trends in absolute barrier errors follow a similar pattern to the TS RMSD observed across different MLIPs. LEFTNet achieves the lowest MAE of 1.83 kcal/mol, while ANI‐1x has the highest MAE of 11.07 kcal/mol, even slightly worse than GFN2‐xTB. These MAEs are higher than the energy MAEs (e.g., 0.81 kcal/mol for LEFTNet) observed on the total validation set (Figure 2b), which can be attributed to two main factors: (1) the barrier is calculated as the energy difference between the TS and reactant, which may accumulate and lead to larger barrier errors, and (2) the absolute barrier error involves comparing MLIP‐optimized geometries to DFT‐optimized ones, which introduces geometry error and results in a higher barrier error. When analyzing the validation set by the number of heavy atoms, which serves as a proxy for system size, we observe a slight increase in error with increasing molecular complexity (Figure S1, Supporting Information). This suggests that the model's scalability with respect to system size may be limited.

The metrics mentioned above can be classified into two categories. Energy and force MAEs, which can be directly obtained from model training and validation, are explicit metrics that are commonly used to judge the quality of MLIPs. On the other hand, success and intended rates, TS RMSD, and barrier MAE, which can only be derived after completing end‐to‐end TS search, are implicit metrics that more accurately reflect the quality of the TS search process. A strong correlation between these two sets of metrics was observed (Figure 2f,g). For example, MLIPs that achieve lower energy and force MAE always yield lower RMSD and reduced barriers for successfully identified TSs. Interestingly, this trend is consistent on both models with the same architectures but trained with different numbers of epochs (Figure 2f) and those converged across different architectures (Figure 2g). However, both the GSM success rates and intended rates saturate at a certain MAE threshold, with an upper bound of GSM success rate of 88% and intended rate of 68%. Below this MAE threshold, although more accurate TS structures can be obtained for TS that are successfully optimized by MLIPs, those unintended TSs remain unintended in spite of the improvement of energy and force MAE. This behavior of saturation also underscores the importance of evaluating MLIPs on an end‐to‐end TS search workflow, as it suggests that further improvements in MLIP accuracy will have diminishing returns in terms of increasing TS search success rate, which is the primary goal of realistic reaction network exploration.

### 2.3 Evaluating MLIPs With Different Force Training and Inference Schemes

**Figure 3:**
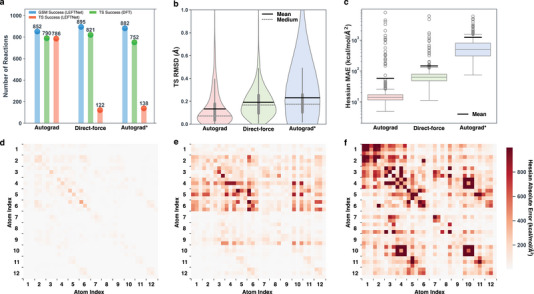
Comparison of MLIP performance under different force training and inference schemes: direct force prediction, energy‐derivative‐based forces (autograd), and training with direct force prediction while applying autograd during inference (autograd*). a) Number of reactions with a converged GSM pathway (blue) and successfully optimized TSs (orange and green). The Hessian‐based TS optimization uses Hessians calculated by the respective LEFTNet schemes (orange) or DFT (ωB97X/6‐31G*, green). b) RMSD between GSM‐obtained TSs and DFT‐optimized TSs. The mean RMSD is indicated by a bold black line, and the median RMSD is shown as a gray dotted line. c) Box‐and‐whisker plots illustrating the mean absolute error (MAE) of Hessians, computed as the derivative of forces from three different schemes, compared to reference DFT‐computed Hessians of GSM‐obtained TSs. d–f) Visualization of absolute errors (AE) between DFT‐computed Hessians and those derived from three schemes. The selected example reaction represents the median Hessian MAE case for the autograd scheme (reaction id: 2357).

Inspired by the observation that the Orb‐v2 model excels in energy and force MAE but yields a low intended rate in TS search, we systematically investigated different force training and inference schemes. Three schemes are considered for the best‐performing MLIP–LEFTNet: direct force prediction, energy‐derivative‐based forces (autograd), and training with direct force prediction while applying autograd during inference (autograd*). The overall TS search success rates, TS quality (TS RMSD), and Hessian quality (Hessian MAE) are compared (**Figure** **3**a–c). Despite the direct‐force and autograd* schemes having slightly higher GSM success rates (>89%), the mean RMSDs of the resulting TSs are 1.2 and 1.8 times higher compared to those obtained by the autograd scheme, respectively (Figure 3a). In addition, the GSM‐level TS structures obtained in the direct force and autograd* schemes suffer at converging to intended reactions, with intended rates of 13.6% and 15.6%, respectively, in line with our previous observation of Orb performance. This can be explained by the extremely high Hessian errors introduced in direct force and autograd* schemes (average Hessian MAE of 144.2 and 1214.6 kcal/mol/Å^2^, Figure 3c). Additionally, while the Hessians computed by the direct‐force scheme exhibit lower errors compared to those from the autograd* scheme, the asymmetric nature of the Hessians derived from direct forces, which lack physical constraints, contributes to a further decrease in the convergence rate. This asymmetry, along with the large Hessian errors, can be seen in a ring opening coupled with a hydrogen transfer reaction, which represents the median Hessian MAE case for the autograd scheme (Figure 3d–f).

To verify the hypothesis that the low intended rate in the direct force and autograd* schemes is caused by their inaccurate Hessian predictions, we further applied DFT‐level TS optimization on all three schemes. Significant improvements in the intended rate were observed for both the direct force and autograd* schemes, with minimal change in the autograd scheme, providing strong evidence in support of our hypothesis (Figure 3a). Notably, the direct‐force scheme identifies the greatest number of GSM pathways, as well as TSs when TS optimization is performed with DFT‐level Hessians. This observation suggests that the direct‐force scheme outperforms the autograd scheme in terms of the number of identified TSs when only accurate forces are required. This highlights an opportunity for further improvement through direct Hessian predictions. By employing a model to directly predict energy, forces, and Hessians, it may be possible to achieve TS search accuracy that surpasses the performance of all three conventional schemes.

### 2.4 Comparison of MLIPs and Generative Models in TS Search

As generative models show promise in bypassing traditional physics‐based TS search process, we compare React‐OT, a state‐of‐the‐art generative model tailored for TS search with its chemically intuitive TS guess and optimal transport, with MLIP. To facilitate its direct comparison to the MLIP‐based approaches, DFT‐level TS optimizations and IRC calculations are performed on initial TS guesses provided by both methods. The MLIP‐based approach, after completing a full round of TS search and verification (Figure 1a–d), with LEFTNet as the best‐performing model, identifies 661 intended TSs and 191 TSs obtained from GSM calculations that turn out to be unintended after TS optimization. These TSs serve as initial guesses for DFT‐level optimizations and IRC calculations (denoted as “LEFTNet+DFT”), leading to 720 intended TSs. Meanwhile, the 960 TS structures generated by React‐OT (treated as GSM success) result in 836 and 913 intended TSs after LEFTNet‐ and DFT‐level calculations, respectively (Figure 1a'‐d, denoted as “ReactOT+DFT”). This observation shows that React‐OT leads to more intended reactions compared to MLIP, with the same model architecture (LEFTNet) being used in both approaches.

**Figure 4:**
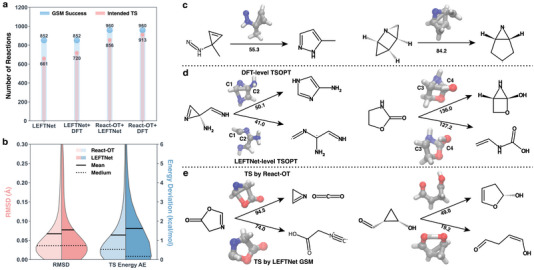
Comparison of MLIPs (LEFTNet) and the diffusion model (React‐OT) in locating TSs for given reactants and products. a) Number of reactions with converged GSM pathways (blue) and intended TSs (red) for four TS search strategies: (1) all steps (Figure 1a‐d) performed at the LEFTNet level; (2) steps a‐b performed at the LEFTNet level, followed by steps c‐d at the DFT level; React‐OT generates the TS (Figure 1a',b'), with steps c‐d performed at (3) the LEFTNet level; and (4) the DFT level. b) Violin plots showing the RMSD (lighter red for React‐OT; darker red for LEFTNet) and absolute errors in TS energies (lighter blue for React‐OT; darker blue for LEFTNet), calculated between the TSs obtained using React‐OT (Figure 1a'‐b') and LEFTNet (Figure 1a–d), relative to DFT‐optimized TSs. For a direct comparison, the TS energy errors are calculated as the difference between DFT‐level single‐point energies on the predicted TSs and the DFT‐optimized TSs. c–e) Selected reactions representing specific scenarios: c) Reactions for which only strategy 4 (React‐OT + DFT) successfully identifies intended TSs. d) TS structures generated by performing step a‐b at the LEFTNet level are subsequently optimized using DFT (strategy 2) and LEFTNet (strategy 1), resulting in an intended TS (upper) and an unintended TS (lower), respectively. e) Starting from TS structures generated by React‐OT and LEFTNet, TS optimizations using LEFTNet (strategy 3 and 1, respectively) yield an intended TS (upper) and an unintended TS (lower). The activation energies calculated by the corresponding strategies are provided in kcal/mol. Atom colors: carbon (gray), nitrogen (blue), oxygen (red), and hydrogen (white).

From the 700 reactions with DFT‐level intended TSs identified by both approaches, the RMSD and energy deviations of the TSs generated by LEFTNet (MLIP) and React‐OT are compared, using DFT‐level TSs as the reference, to assess the accuracy and reliability of the initial TS guesses produced by each method. Reactions with TSs showing deviations greater than 100 kcal/mol (14 in total, all from LEFTNet) are excluded, reducing the dataset from 700 to 686. The TSs generated by React‐OT are superior in geometry, but comparable in energy predictions when compared with those from LEFTNet (Figure 4b). The mean RMSD for React‐OT is 0.067 Å, better than LEFTNet (0.077 Å), while the median RMSDs are both 0.036 Å. Regarding energy deviations, among 700 reactions, LEFTNet exhibits several outliers: 14 TSs with deviations greater than 100 kcal/mol and 44 TSs with deviations over 10 kcal/mol, which accounts for the significant gap in mean and median energy errors. In contrast, React‐OT shows no TSs with deviations larger than 100 kcal/mol and only 13 TSs with deviations exceeding 10 kcal/mol. After excluding outliers with energy errors greater than 10 kcal/mol, the MAEs for React‐OT and LEFTNet are 1.03 kcal/mol and 0.77 kcal/mol, respectively. While React‐OT performs slightly worse in TS energy prediction, likely because it does not utilize energy information during training, it nonetheless provides a more reliable and robust TS prediction overall. This is further supported by the fact that TSs generated by LEFTNet (step a‐b) require slightly more gradient calls in the DFT‐level TS optimization (Figure S6, Supporting Information). Additionally, to reduce the dependency on DFT calculations, React‐OT can be combined with MLIPs to filter out potentially “unintended” reactions and improve barrier prediction accuracy. The same TS search workflow (Figure 1a'‐d) is applied, but with TS optimization and IRC calculations performed using LEFTNet instead of DFT (denoted as “ReactOT+LEFTNet”). This approach identifies 856 intended TSs (Figure 4a), which is even more than the LEFTNet+DFT approach, highlighting its potential for real‐world applications where DFT calculations are costly.

Here we demonstrate specific reactions to understand the differences in TS structures obtained by LEFTNet and React‐OT, as well as the TS optimization performed by LEFTNet and DFT. Three types of reactions emerge from this comparison: (1) reactions where only the ReactOT+DFT workflow identifies intended TSs (28 in total), (2) reactions where LEFTNet+DFT identifies intended TSs but LEFTNet alone does not (77 in total), and (3) reactions where ReactOT+LEFTNet locates intended TSs but LEFTNet itself does not (219 in total). For each of these types, two reactions are randomly selected for detailed analysis (**Figure** **4**c–e). In the first scenario, it is commonly observed that either the reactant or product has relatively high energy due to the presence of zwitterionic species (Figure 4c, left) or di‐radical species (Figure 4c, right), both of which pose significant challenges for MLIP learning. In the second scenario, starting from the same TS initial guess, TS optimizations performed by DFT and LEFTNet result in TS structures with key bond length differences. For example, the distance between two carbon atoms (C1‐C2 and C3‐C4 in Figure 4d) dictates whether the reaction undergoes a ring‐closing or ring‐opening process, with one pathway being intended and the other unintended, respectively. Similarly, in the third scenario, a subtle difference in the key bond lengths between TS initial guesses provided by React‐OT and LEFTNet leads to distinct optimized TS structures, resulting in different reaction pathways (Figure 4e).

### 2.5 A Realistic Reaction Network: γ‐Ketohydroperoxide Decomposition

**Figure 5:**
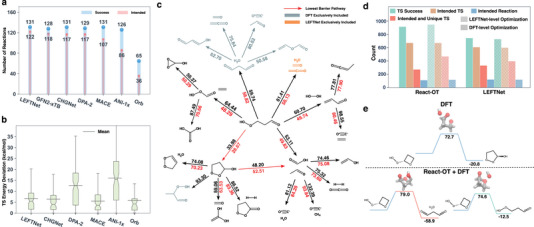
Testing six MLIPs, GFN2‐xTB, and React‐OT on a reaction network problem. a) Number of reactions with at least one TS conformation that passes all four steps in the TS search workflow (denoted as success, blue) and with at least one intended TS (red), over all attempted reaction conformations. b) Absolute deviations between the lowest TS energy across all intended TS conformations calculated by MLIPs and DFT. c) The reaction network generated at ωB97X/6‐31G* and LEFTNet levels of theory. Activation energies for the corresponding pathways are provided in kcal/mol, with black representing DFT‐level energies and red for LEFTNet‐level energies. Reactions exclusively included in the DFT‐level and LEFTNet‐level reaction network are highlighted in dark blue and orange, respectively. d) Number of different types of TSs obtained by four schemes: React‐OT+LEFTNet, React‐OT+DFT, LEFTNet, and LEFTNet+DFT. e) Example of a reaction where the DFT‐based approach identifies an intended TS (upper), while the React‐OT+DFT approach identifies two unintended TSs (lower).

The MLIPs and React‐OT are further benchmarked on a reaction network problem–γ‐ketohydroperoxide (KHP) decomposition network, which represents a real‐world application and has been benchmarked in many recent studies.^[^ ^64^, ^65^, ^78^ ^]^ Unlike benchmarking on a dataset of individual reactions, reaction network exploration requires correctly ranking the barriers of reactions originating from the reactant and each intermediate (node in the reaction network), as well as identifying important multi‐step pathways. The original set of 131 reactions from a previous study on the KHP reaction network was collected,^[^ ^65^ ^]^ which was then recomputed using YARP for the purpose of making the level of theory consistent. For each reaction, up to ten reaction conformations were sampled, resulting in a total of 1065 reaction conformations. 129 out of 131 reactions have at least one intended TS at the ωB97X/6‐31G* level, with the lowest energy TS of each reaction serving as the ground truth. These 1065 reaction conformations were calculated using the same TS search workflow (Figure 1) with six MLIPs trained on the Transition1x dataset, GFN2‐xTB, and React‐OT. For each reaction, if at least one reaction conformation results in a converged and intended TS, the reaction is counted as “success” and “intended”, respectively (**Figure** **5**a). The result counting the number of individual conformations is provided in Figure S7 (Supporting Information).

LEFTNet consistently identifies the highest number of intended reactions (122), followed by GFN2‐xTB (118), CHGNet (117), DPA‐2 (117), and MACE‐OFF23 (107). The Orb‐v2 model identifies the lowest number of both success and intended reactions (36), due to the direct‐force prediction scheme. Although many MLIPs achieve high intended rates in the KHP reaction network, a notable discrepancy is observed in the energy evaluations (Figure 5b), where the lowest TS energy predicted by each model is compared to the DFT reference value. On the subset of intended reactions, even the best‐performing model (MACE‐OFF23) exhibits a MAE greater than 5 kcal/mol, which is significantly larger than the energy errors observed on the Transition1x validation set (less than 2 kcal/mol). Moreover, although DPA‐2 achieves a relatively high intended rate, the corresponding MAE for the lowest TS energy is considerably high (12.6 kcal/mol), suggesting that while the model effectively identifies intended TSs, it struggles to accurately predict their energy profiles. Notably, these two metrics are both critical in reaction network exploration, as constraints are typically applied to limit the expansion of each node and prevent the network from growing explosively. A simple constraint that can be set is that intermediates are only further explored if they are connected by the lowest five barriers, which helps maintain computational efficiency and ensures the network remains manageable.^[^ ^9^, ^79^ ^]^ As such, to explore the reaction network both efficiently and accurately, MLIPs must not only identify the intended reactions but also rank them with high accuracy to prioritize the most relevant pathways. Reaction networks explored by DFT and LEFTNet are visualized and compared under such constraint (Figure 5c). LEFTNet correctly identifies the top‐ranked intermediate (the five‐member ring) and four out of the top five first‐step products. The only missing top‐five reaction (denoted in dark blue in Figure 5c), a dehydration reaction, is ranked sixth in barrier height by LEFTNet, with a predicted barrier of 56.82 kcal/mol. In contrast, a high‐barrier decomposition reaction is ranked fifth (denoted in orange). The second‐step reactions derived from the correctly ranked four intermediates are similar, with only one missing reaction: a ring‐opening reaction with a relatively high barrier (denoted in dark blue). Notably, the most important decomposition pathway – the cyclic reaction followed by decomposition into acetaldehyde and formic acid, known as the Korcek mechanism – is correctly identified by LEFTNet as the most kinetically favorable reaction. Reaction networks explored by other MLIP and React‐OT are provided in Figures S8–S13 (Supporting Information).

To further compare React‐OT and LEFTNet on the reaction network problem, four different schemes, developed for comparing MLIP and generative models, were applied to the 1065 reaction conformations, isolating the effects of TS initial guesses and subsequent optimization. These schemes combine different TS guess generation methods (LEFTNet vs. React‐OT) and levels of optimization theory (LEFTNet vs. DFT). Instead of focusing solely on the number of reactions with at least one intended TS conformation (blue box in Figure 5d), each TS conformation was counted and categorized into three types: converged TSs (green), intended TSs (brown), and unique intended TSs (red), with redundant TSs removed based on energy deviations smaller than 0.1 kcal/mol (Figure 5d). The React‐OT+DFT scheme identifies the highest number of all three types of TS conformations and slightly fewer reactions with at least one intended TS (119), indicating that it is the overall most accurate scheme. There is a significant drop in the number of intended and unique TS conformations after switching from DFT‐level to LEFTNet‐level optimization on React‐OT generated TSs (471–274), leading to 111 intended reactions identified. In contrast, for the TS initial guesses generated by LEFTNet, the number of intended and unique TS conformations, as well as the number of intended reactions, are not significantly affected by the choice of theory level during TS optimization (Figure 5d, right part). This observation indicates that the slightly poorer performance of the React‐OT+LEFTNet scheme may be attributed to the generalizability limitations of LEFTNet or the inconsistency originating from the mixed use of multiple ML models. Although the focus of this study was primarily on intended reactions for a consistent comparison across methods, identifying unintended reactions offers valuable insights into new and unexpected chemistry. Generative modeling is exactly a path toward this goal. For example, in a reaction where React‐OT+DFT fails to identify any intended TS conformation (Figure 5e), two unintended reactions are revealed: one with a similar barrier (Figure 5e, bottom right) and another with a significantly more thermodynamically favorable product (bottom left), which represents potentially new reaction mechanisms that are not initially proposed.

## 3 Conclusion

Accelerating the search for TS and the prediction of activation energy is important to enable efficient exploration of reaction networks and facilitate high‐throughput screening of complex chemical systems.^[^ ^3^, ^4^, ^6^, ^12^ ^]^ In this study, we conducted a systematic evaluation of ML approaches for transition state (TS) search, including both MLIPs and generative models. We found that all pre‐trained universal MLIPs struggle with a low success rate of locating intended TSs, highlighting the need to incorporate more reactive data into the pre‐training process. Among the MLIPs evaluated, LEFTNet achieves the best performance after fine‐tuning on the Transition1x dataset, demonstrating strong generalization to both in‐field (withheld validation) and out‐of‐field reactions (e.g., the KHP network). Meanwhile, React‐OT outperforms MLIPs on the validation set, as it does not require an accurate description of the entire region around the minimum energy path (MEP). Notably, combining React‐OT with LEFTNet offers a promising strategy that leverages the strengths of both models: the robustness of React‐OT in generating initial TS guesses and the efficiency of LEFTNet in instant TS refinement and barrier estimation, thereby reducing reliance on expensive DFT calculations in automated reaction network exploration.

To further assess MLIP development, we explored different training and inference schemes, such as autograd versus direct‐force approaches. Though Hessians are not conventionally required in most molecular dynamics (MD) simulations, where the primary objective is to accurately predict energies and forces, in the context of TS optimization, Hessians play a critical role in guiding the search along the correct curvature of the potential energy surface. By comparing three different LEFTNet training and inference schemes, we illustrate the importance of accurate and physically realistic Hessians, underscoring the value of including Hessians during MLIP training, despite their higher computational cost. Notably, while direct‐force prediction is effective at reducing force error, the associated symmetry‐breaking errors in the predicted Hessians significantly degrade TS search performance. This highlights the potential of extending MLIPs to directly predict Hessians, in addition to energies and forces, as a strategy for improving accuracy in TS localization. Furthermore, our results show that incorporating first‐order derivatives of energy (i.e., forces) into the loss function substantially improves Hessian prediction accuracy, suggesting that integrating second‐order information such as Hessians or related spectral features (e.g., vibrational frequencies) could further enhance model performance in reactivity modeling.

Finally, by comparing traditional energy and force mean absolute errors (MAEs) with task‐specific, end‐to‐end metrics, we find that MAEs alone are insufficient for evaluating model performance in chemical reactivity modeling. While MAEs are useful for assessing general accuracy, they do not always align with the model's ability to locate transition states or identify intended reactions. To provide a more comprehensive evaluation, we introduce a set of end‐to‐end metrics, including success rate, intended rate, TS RMSD, and barrier error, that better reflect the practical effectiveness of ML models in TS search. The success and intended rates measure a model's ability to identify valid TSs and avoid missing important reactions, while TS RMSD and barrier errors assess the quality of the predicted TSs, which is critical for pathway ranking and mechanistic interpretation. Although MAEs often exhibit trends consistent with TS RMSD and barrier error, they can diverge from success and intended rates due to more complex factors such as Hessian quality. These findings highlight the importance of using multiple task‐specific metrics and offer guidance for the development and evaluation of ML models in reaction modeling.

We also acknowledge several limitations of this study. First, the generalizability of different MLIP architectures requires further evaluation, as the current results are constrained by the coverage of the Transition1x dataset, in which molecules have up to seven heavy atoms and are composed of only C, N, O, and H elements. Additionally, with an average of 1000 data points sampled per reaction, there is a risk of redundancy in the sampled geometries, and some regions of the reaction space may be underrepresented, especially those that deviate from the DFT‐level MEP. To unlock the full potential of MLIPs and diffusion models, a more diverse set of reactive datasets is essential, such as those involving solution‐based reactions.^[^ ^80^ ^]^ Furthermore, the inherent connections between MLIPs and diffusion models have not been fully explored in this study. Notably, the diffusion model can be viewed as an interatomic potential under the harmonic approximation.^[^ ^47^, ^81^ ^]^ In this context, the LEFTNet architecture is employed both as the scoring network for React‐OT and as the DFT surrogate in MLIPs, with the two models trained for different objectives. A promising direction for future work could involve transferring weights learned from MLIPs, which are trained on larger datasets for energy and force prediction, to fine‐tune diffusion models like React‐OT, potentially improving performance. In the end, while the evaluation among different MLIPs and diffusion models mainly focuses on the intended rate, we note that unintended reactions may be critical for uncovering unknown mechanisms and discovering new chemical reactions, as evidenced in Figure 5. A more systematic study on unintended reactions would be insightful after a sampling scheme that seeks the synergy of MLIPs and diffusion models is established.

In summary, this work sets the ground for systematically evaluating and comparing various TS search approaches, especially as more extensive and varied reactive datasets become available, alongside the emergence of more advanced MLIPs and generative models. By broadening the scope of reactive systems and incorporating advancements in both machine learning and computational chemistry, this evaluation will continue to be essential for enhancing model accuracy and deepening our understanding of complex reactions.

## 4 Experimental Section

### Details on TS Search Algorithms

TS search in this study consists of two primary steps: GSM^[^ ^72^ ^]^ and a Hessian‐based TS optimization determined by the RS‐I‐RFO.^[^ ^73^ ^]^ The GSM is performed using the pygsm package,^[^ ^82^ ^]^ where various MLIPs are incorporated into the ASE^[^ ^83^ ^]^ calculator for energy and force evaluations. Default convergence parameters, including nine nodes, the climbing image setting, and the translation‐rotation‐internal coordinate system, are applied consistently across all calculations.

In addition to GSM, NEB method^[^ ^84^ ^]^ was also tested as an alternative approach to construct the MEP. However, since NEB calculations heavily rely on an initial guess, there is a higher risk of calculating high‐energy regions. Consequently, the convergence rate for NEB was found to be much lower than that of GSM when using pre‐trained MLIPs and was thus discarded.

The RS‐I‐RFO optimization is carried out using the pysisyphus package,^[^ ^85^ ^]^ with a trust radius set to 0.2Å, the convergence threshold set to the default Gaussian parameters, and the maximum number of optimization steps limited to 50. If the calculation is performed using MLIPs, Hessians are recalculated at every step. If DFT is used for the optimization, Hessians are recalculated every three steps.

IRC performed to verify the optimized TS is also carried out using the pysisyphus package. IRCs are integrated in mass‐weighted coordinates with the default “EulerPC” integrator. The resulting endpoints of the IRC integration are further optimized to stationary points. The comparison of IRC endpoints and input reactants and products is based on the adjacency matrix computed by the TAFFI package.^[^ ^86^ ^]^

### Computational Details

All calculations in this study were carried out on the Volcengine. MLIP‐based simulations were performed as bundled single‐core jobs on a CPU machine with 32 effective cores, Intel Cascade Lake CPUs (2.40 GHz), and 128 GB of memory. DFT calculations utilized GPU4PYSCF^[^ ^87^ ^]^ as the quantum chemistry engine and were executed on A30 GPU cards.

### Dataset

The Transition1x dataset consists of 10,073 reactions that are originally sampled by Grambow et al.^[^ ^75^ ^]^ These reactions are re‐computed by CI‐NEB at the ωB97X/6‐31G* level, resulting in 9.6 million geometries. The same data split of reactions used in the React‐OT study was applied,^[^ ^31^ ^]^ with 9000 and 1073 reactions allocated to the training and validation set, respectively. After performing TS optimization and IRC calculations at the same level, 113 reactions were identified as unintended and subsequently removed from the validation set for the TS search tasks.

The original KHP decomposition network was established using YARP.^[^ ^65^ ^]^ While the reaction exploration adhered to a “breaking two bonds and forming two bonds” rule, the IRC calculations uncovered unintended reactions that involve more complex chemistry, such as “breaking three bonds and forming three bonds.” Initial exploration was performed at the M052X‐D3/def2‐SVP level of theory. To ensure consistency with the Transition1x dataset, these 131 reactions were re‐calculated at ωB97X/6‐31G* level. To maximize the identification of intended TSs and increase the probability of finding the most stable TS, a reaction conformational sampling strategy was applied.^[^ ^88^ ^]^ This strategy sampled both the reactant and product sides, generating up to ten reaction conformations per reaction. The subsequent calculations followed the regular YARP recipe.

### MLIP Models

In this study, we trained six MLIPs on the Transition1x dataset. The selected models, including LEFTNet, MACE, Orb, CHGNet, ANI‐1x, and DPA‐2, exhibit diverse architectures. Among them, ANI‐1x and DPA‐2 are descriptor‐based models that utilize fixed rules to encode the environment of atoms. The encoded information is then used as input for a standard feed‐forward neural network. LEFTNet, MACE, Orb, and CHGNet are graph neural network‐based models that treat molecules as undirected graphs. In these models, atoms are represented as nodes while pair‐wise interactions between atoms are treated as edges. Through message‐passing, these models can effectively capture complex chemical interactions.

*ANI‐1x*. ANI model is based on modified Behler and Parrinello symmetry functions(BPSFs)^[^ ^89^ ^]^ and their high‐dimensional feed‐forward neural network potential model. The modified BPSFs are used to compute atomic environment vectors as local descriptors. The ANI‐1x model is an ensemble of eight base models that were trained using active learning on the ANI‐1x dataset.^[^ ^39^, ^40^ ^]^

*CHGNet*. CHGNet is an invariant GNN designed for crystal structures. The model provides atomic charge via magnetic moment prediction using only the nuclear positions and atomic identities as input, allowing the study of charge distribution in atomistic modeling. CHGNet is pretrained on the energies, interatomic forces, stresses, and magnetic moments derived from the DFT calculations in the MPTrj dataset,^[^ ^67^ ^]^ which encompasses more than 1.5 million atomic configurations for inorganic materials.

*DPA‐2*. DPA‐2 is an invariant GNN implemented within the DeePMD‐kit framework.^[^ ^90^ ^]^ Designed as a large atomic model, DPA‐2 is specifically tailored to integrate and simultaneously train on datasets from various disciplines, encompassing diverse chemical and materials systems across different research domains. The DPA‐2 model leverages 28 distinct datasets to pre‐train a unified descriptor, which can be efficiently fine‐tuned in downstream tasks.^[^ ^43^ ^]^

*LEFTNet*. LEFTNet is a SE(3) equivariant (and reflection anti‐equivariant) GNN that efficiently implements local 3D substructure encoding (LSE) and frame transition encoding (FTE). LSE ensures an invariant realization, while FTE is crucial for maintaining equivariance throughout the feature transfer process during the transition from local to global representations.^[^ ^48^ ^]^

*MACE*. MACE is an E(3) EPNN that efficiently combines higher‐order equivariant message passing with many‐body messages.^[^ ^45^ ^]^ It uses symmetric features to describe the chemical environment and employs a many‐body expansion to parametrize these features. MACE‐OFF23 is a foundation model based on MACE, pre‐trained on chemical and biomolecular datasets that encompass a total of 951 813 training samples and 8 element types (H, C, N, O, P, S, Br, I).^[^ ^70^ ^]^

*Orb*. The backbone of Orb is an invariant GNN augmented with a smoothed graph attention mechanism, where messages between nodes are updated based on both attention weights and distance‐based cutoff functions. The model is trained in two phases: first, it is trained as a scoring function in a Denoising Diffusion model using a dataset of ground state materials. Second, the base model initializes a MLIP, which is trained in a supervised manner to predict energy, forces, and stress of MPTrj and Alexandria datasets.^[^ ^71^ ^]^

Training details and learning curves of each model are provided in Section S2 (Supporting Information).

## Author Contributions

Q.Z. and Y.H. contributed to methodology, software, validation, investigation, data curation, original draft writing, review and editing, and visualization. D.Z. and P.Z. were involved in data curation, as well as review and editing. J.W. contributed to software, visualization, and review and editing. T.C. participated in original draft writing and review and editing. Y.C. contributed to software and review and editing. B.Y. and H.J. were involved in review and editing, with H.J. also providing funding. C.D. was responsible for conceptualization, methodology, investigation, original draft writing, review and editing, visualization, and funding.

## Conflict of Interest

The authors declare no conflict of interest.

## Supporting information

Supporting Information

## Data Availability

Code and data will be available as an open‐source repository on Github.
