# Supplementary material for: Harnessing Machine Learning to Enhance Transition State Search with Interatomic Potentials and Generative Models
Source: Adv Sci (Weinh). 2025 Jul 13;12(34):e06240. doi: 10.1002/advs.202506240 (PMC12442704; doi:10.1002/advs.202506240)
Supplement: Supplementary file 1 — Supporting Information [file ADVS-12-e06240-s001.pdf]

ADVANCED SCIENCE
Open Access

Supporting Information

for *Adv. Sci.*, DOI 10.1002/advs.202506240

Harnessing Machine Learning to Enhance Transition State Search with Interatomic Potentials and Generative Models

Qiyuan Zhao, Yunhong Han, Duo Zhang, Jiaxu Wang, Peichen Zhong, Taoyong Cui, Bangchen Yin, Yirui Cao, Haojun Jia* and Chenru Duan**

Harnessing Machine Learning to Enhance Transition State Search with Interatomic Potentials and Generative Models Supporting Information

Qiyuan Zhao,[*,†,@] Yunhong Han,[†,@] Duo Zhang,[‡,¶,§] Jiaxu Wang,[†] Peichen Zhong,[‖] Taoyong Cui,[†] Bangchen Yin,[⊥] Yirui Cao,[†] Haojun Jia,[*,†] and Chenru Duan[*,†,#]

†*Deep Principle Inc., Cambridge, MA, 02139*

‡*AI for Science Institute, Beijing 100080, P. R. China*

¶*DP Technology, Beijing 100080, P. R. China*

§*Academy for Advanced Interdisciplinary Studies, Peking University, Beijing 100871, P. R. China*

‖*Bakar Institute of Digital Materials for the Planet, UC Berkeley, California 94720, United States*

⊥*Department of Chemistry, Tsinghua University, Beijing 100084, China*

#*Frontiers Science Center for Transformative Molecules, School of Artificial Intelligence, Shanghai Jiao Tong University, Shanghai 200240, China*

@*These authors contributed equally to this work.*

E-mail: zhaoqiyuan@deepprinciple.com; haojunjia@deepprinciple.com; duanchenru@gmail.com

1 Abbreviation

The following is the list of abbreviations utilized in the main paper.

- TS: Transition state.
- ML: Machinea learning.
- DFT: Density functional theory.
- MLIPs: Machine learning interatomic potentials.
- PES: Potential energy surface.
- MEP: Minimun energy path.
- EGNNs: Equivariant graph neural networks.
- OA-ReactDiff: Object-aware SE(3) GNN for generating sets of 3D molecules in elementary reactions under the diffusion model
- React-OT: Optimal Transport approach to generate TSs of an elementary Reaction
- GSM: Growing string method.
- IRC: Intrinsic reaction coordinate.
- RMSD: Root-mean-square deviation.
- MAE: Mean absolute error.
- NEB: Nudged-elastic band.
- CI-NEB: Climbing image nudged elastic band method.
- xTB: eXtended Tight Binding
- KHP: γ-ketohydroperoxide
- RS-I-RFO: Restricted-step rational-function-optimization.

2 Details on model training.

LEFTNet and CHGNet were trained from scratch, while MACE, Orb, and ANI-1x models were fine-tuned from the MACE-OFF23-medium model, Orb-v2 model, and original ANI-1x model, respectively. DPA-2 was fine-tuned using the 26-head pre-trained version, excluding the transition-1x head, with “Drug” head being selected for this task. Two additional attempts were made but resulted in worse performance and were thus not reported. First, fine-tuning CHGNet, which was originally trained for inorganic materials, led to significantly worse energy and force predictions compared to training from scratch. Second, the ANI-1xnr model, which was trained on condensed phase simulations, performed poorly in gas-phase reactions when compared to ANI-1x, both before and after fine-tuning on the transition-1x dataset. Hyper-parameters of these models are provided below. All models are trained using MSE loss, with different weighting ratios between energy (e) and forces (f).

Table S1: Training details for different MLIPs.

Methods	pre-trained model	loss(f:e)	learning rate	batch size
LEFTNet	from scratch	100:1	1e-4	32
MACE	MACE-OFF23-medium	25:1	1e-3	32
Orb	Orb-v2	5:1	1e-4	64
CHGNet	from scratch	10:1	1e-3 $\rightarrow$ 5e-4	128
ANI	ANI-1x	10:1	step (γ=0.8, 100)	128
DPA-2	DPA2-26heads	decay	lr decay	20

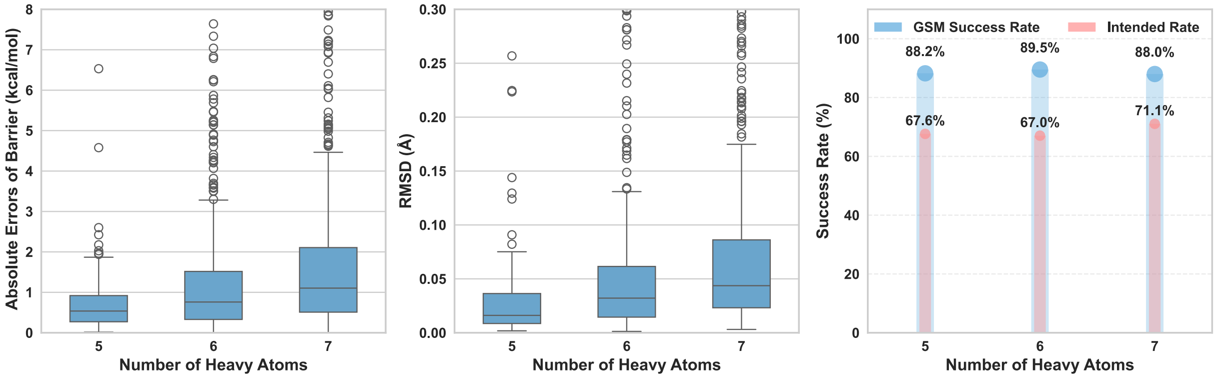

Figure S1: Comparison of some implicit metrics (absolute errors of barrier, TS RMSD, and intended success rate) across different system sizes.

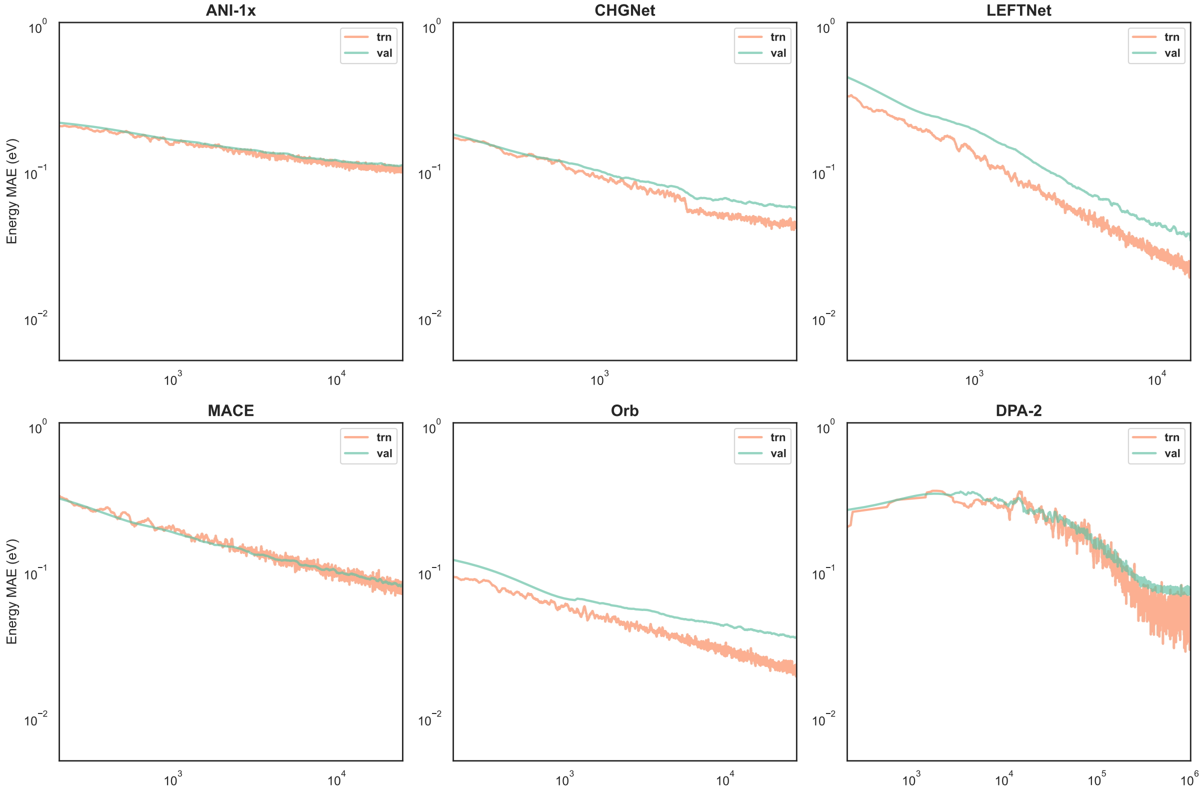

Figure S2: Learning curve of energy MAE for six MLIPs with increasing training steps.

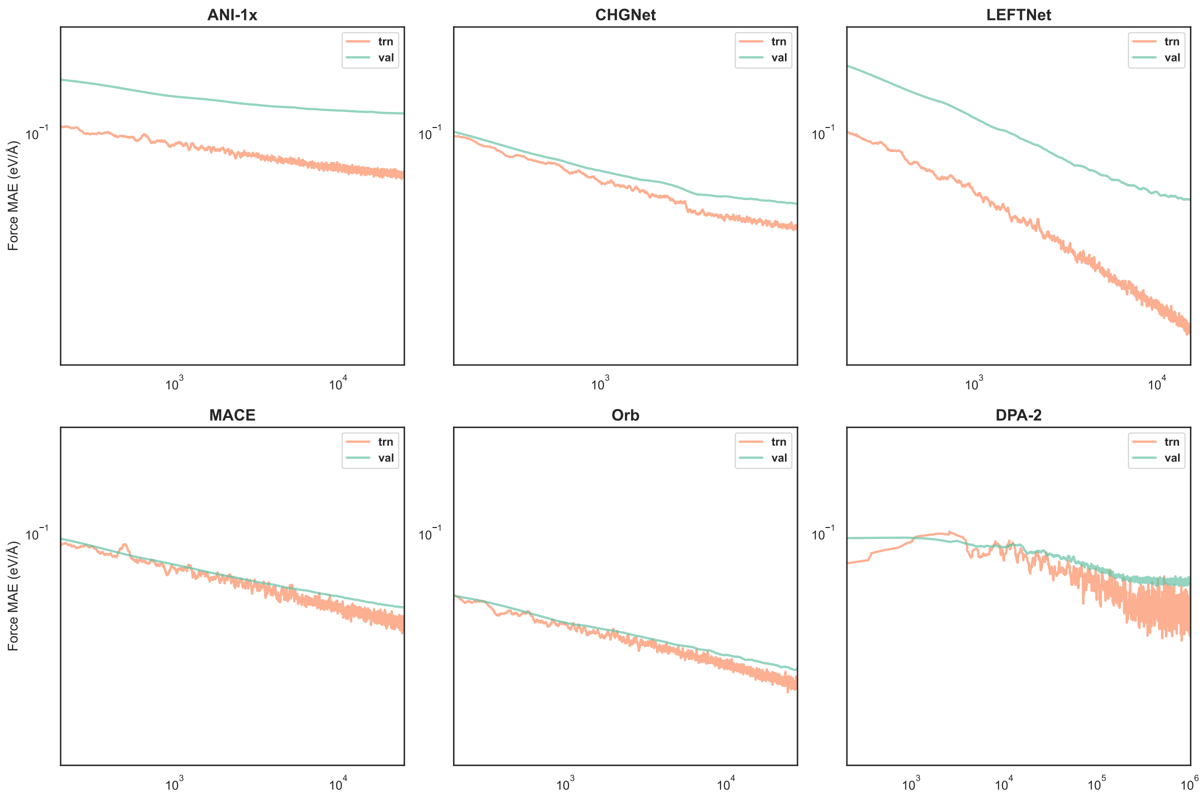

Figure S3: Learning curve of force MAE for six MLIPs with increasing training steps.

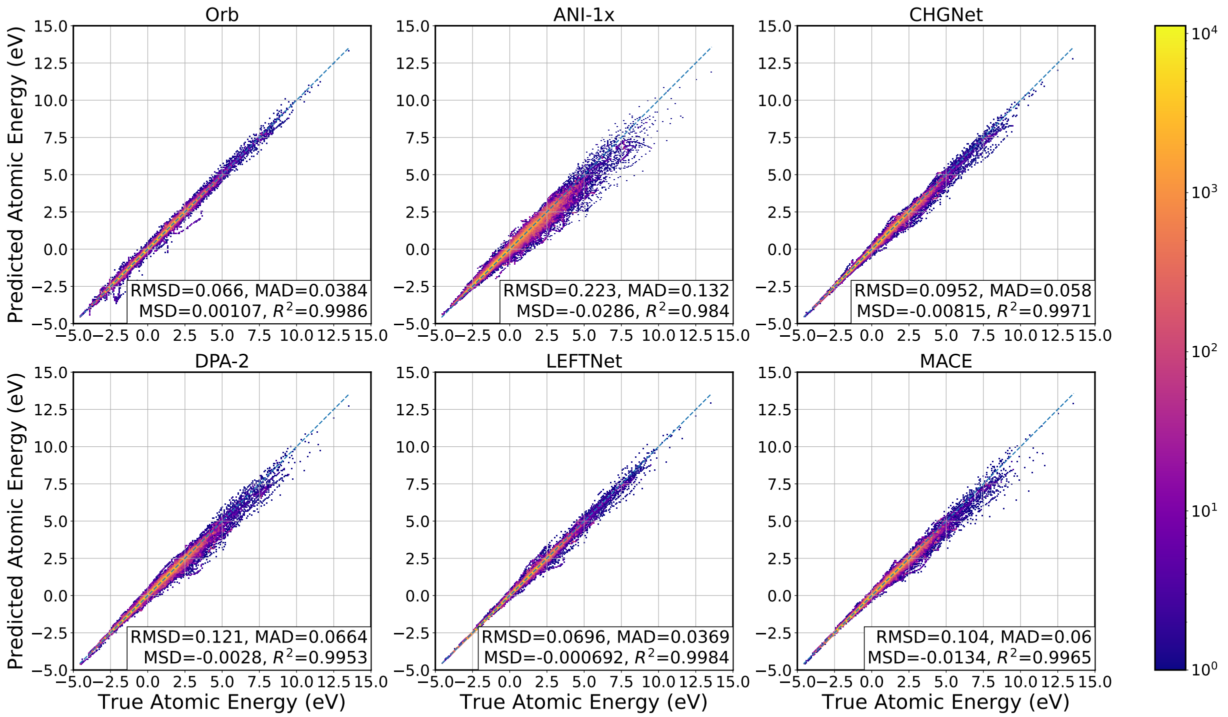

Figure S4: Correlation plots showing the predicted versus true atomic energies for six MLIPs.

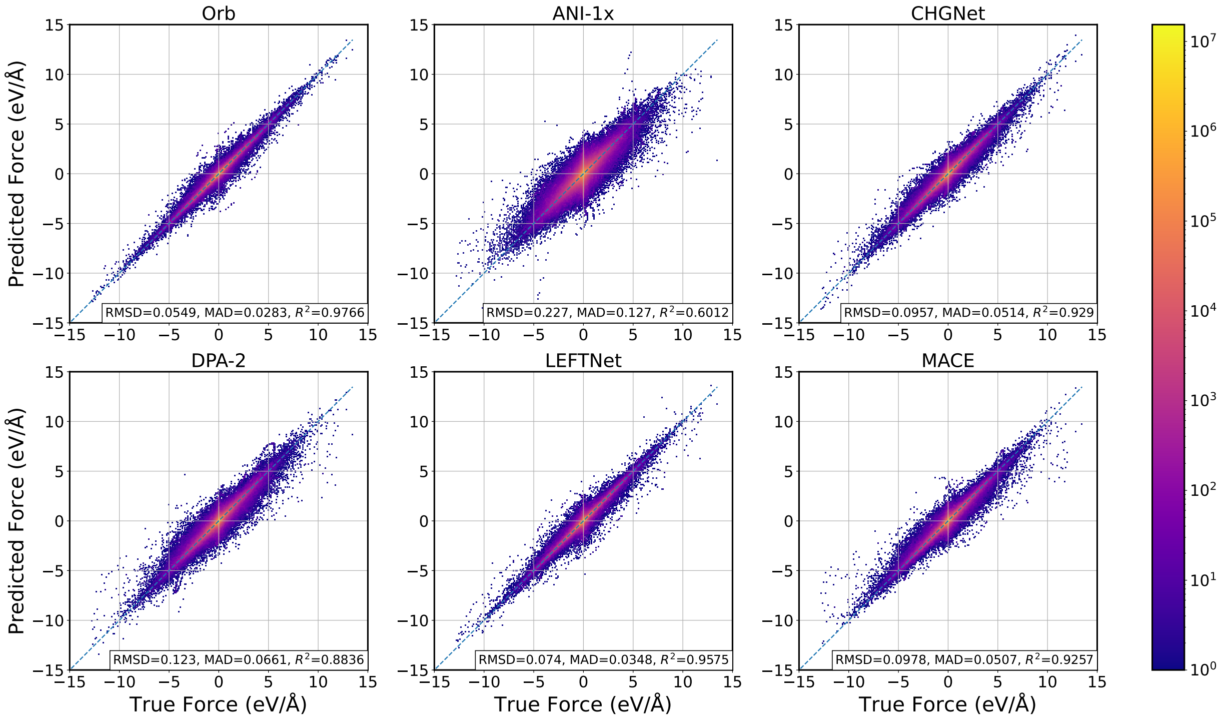

Figure S5: Correlation plots showing the predicted versus true forces for six MLIPs.

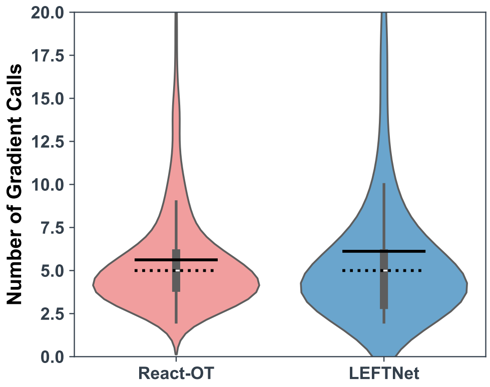

Figure S6: Comparison of number of DFT-level gradient calls in TS optimizaions starting from TS initial guesses provided by React-OT (red) and LEFTNet (blue). Solid line represent the mean values.

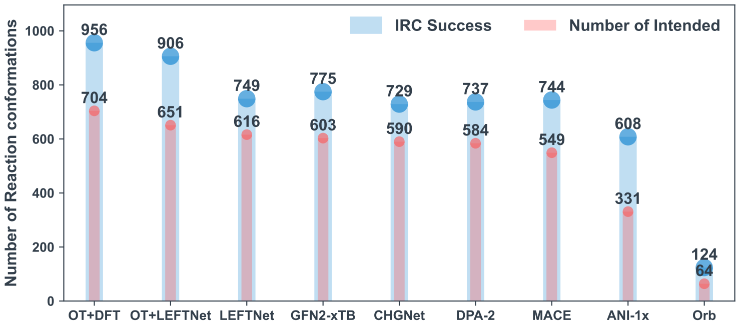

Figure S7: Number of reaction conformations that pass all four steps in the TS search workflow (denoted as success, blue) and turn out to be intended (red).

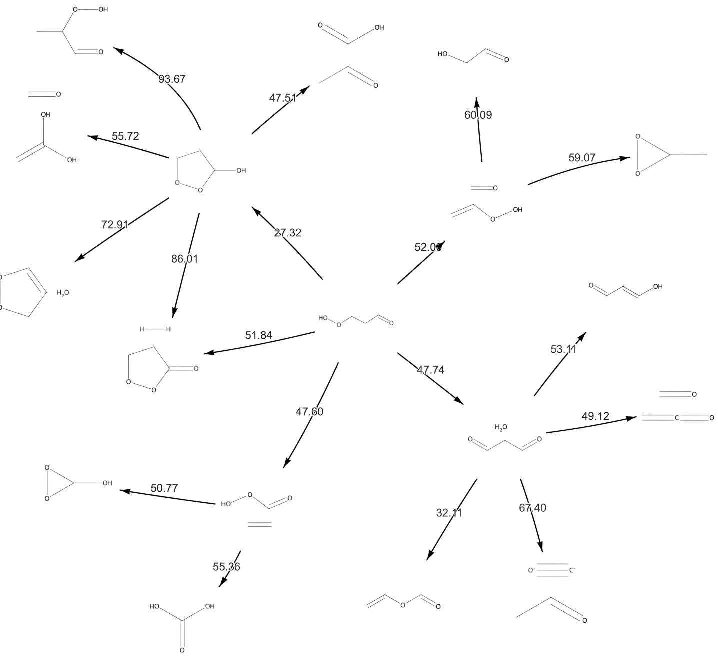

Figure S8: The network is generated using YARP with ANI-1x as computational engines. Activation energies for the corresponding pathways are provided in kcal/mol.

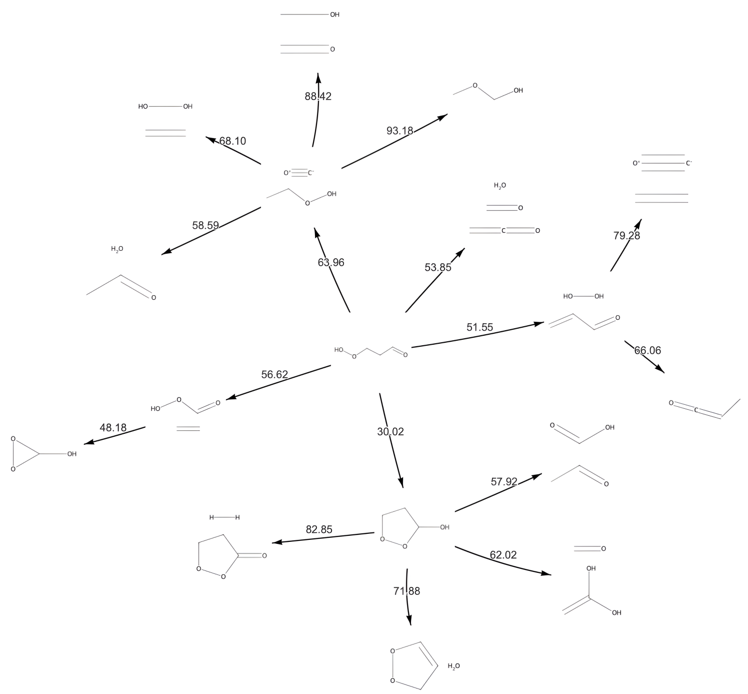

Figure S9: The network is generated using YARP with CHGNet as computational engines. Activation energies for the corresponding pathways are provided in kcal/mol.

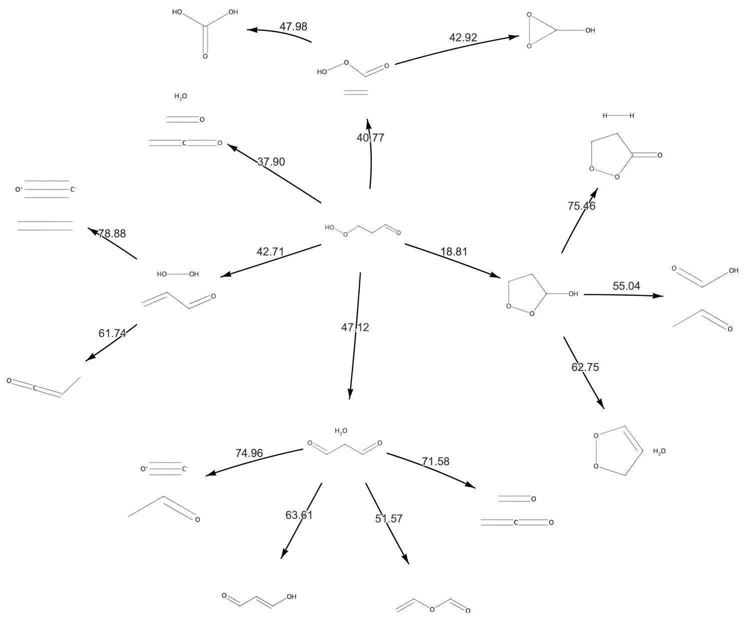

Figure S10: The network is generated using YARP with DPA-2 as computational engines. Activation energies for the corresponding pathways are provided in kcal/mol.

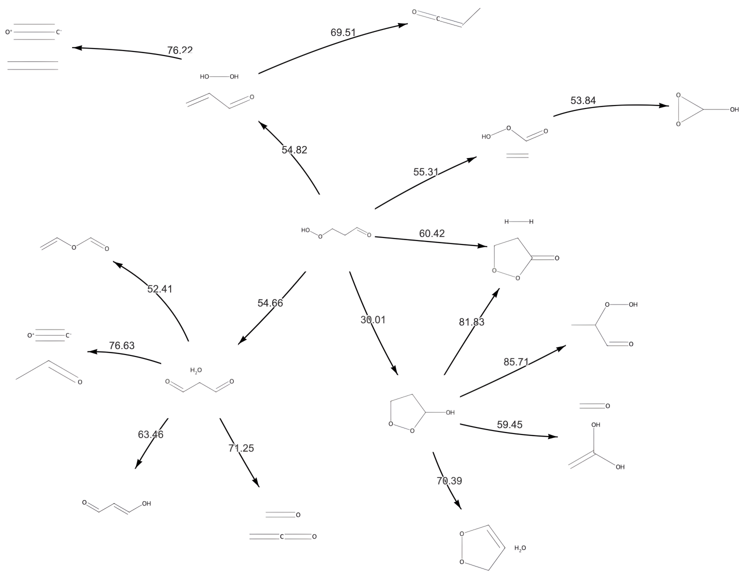

Figure S11: The network is generated using YARP with MACE-OFF23 as computational engines. Activation energies for the corresponding pathways are provided in kcal/mol.

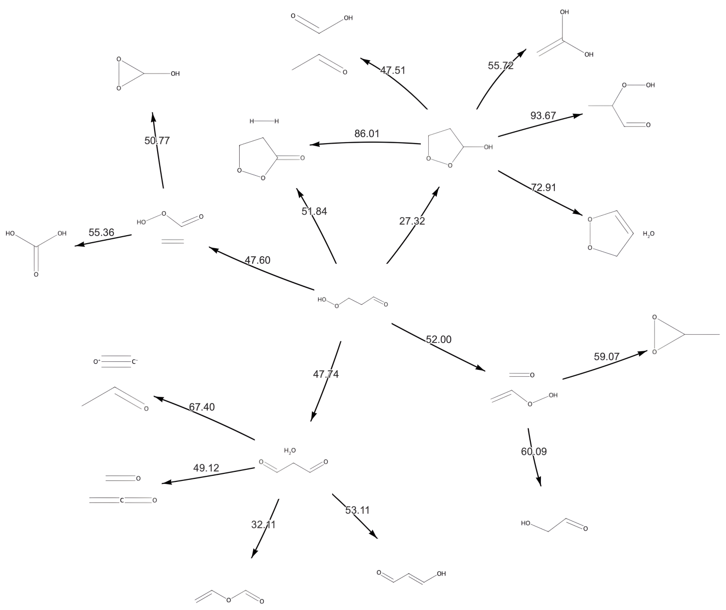

Figure S12: The network is generated using YARP with GFN2-xTB as computational engines. Activation energies for the corresponding pathways are provided in kcal/mol.

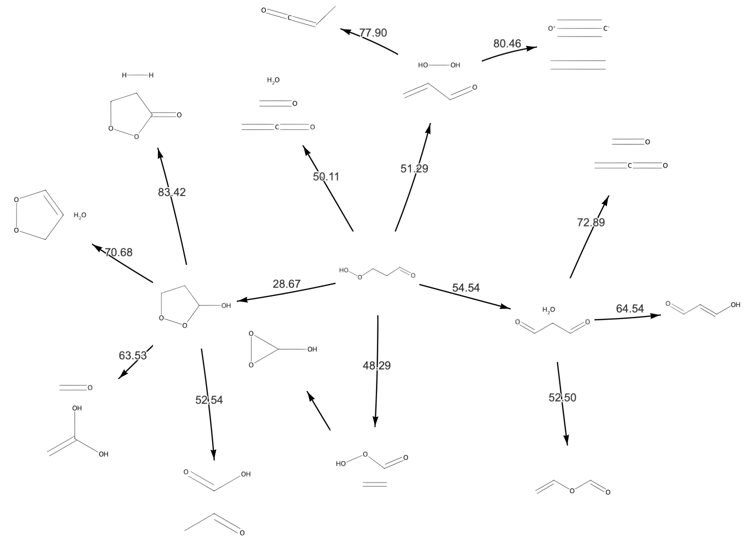

Figure S13: The network generated by React-OT+LEFTNet scheme. Activation energies for the corresponding pathways are provided in kcal/mol.
